# An Assessment of the Diversity and Seasonal Dynamics of Small- and Medium-Sized Mammals in Pittachhara Forest, Bangladesh, Using a Camera Trap Survey

**DOI:** 10.3390/ani14243568

**Published:** 2024-12-10

**Authors:** Raf Ana Rabbi Shawon, Md. Matiur Rahman, Md Mehedi Iqbal, Mahfuz A. Russel, Junji Moribe

**Affiliations:** 1 Laboratory of Wildlife Resources, Gifu University, 1-1 Yanagido, Gifu 501-1193, Japan; rafana.shawon@gmail.com; 2 Laboratory of Food and Environmental Hygiene, Gifu University, 1-1 Yanagido, Gifu 501-1193, Japan; matiur.vetmed@gmail.com; 3 Department of Medicine, Sylhet Agricultural University, Sylhet 3100, Bangladesh; 4 Atmosphere and Ocean Research Institute, The University of Tokyo, 5-1-5 Kashiwanoha, Kashiwa, Chiba 277-8564, Japan; mehedi.imsf@gmail.com; 5 Pittachhara Forest and Biodiversity Conservation Initiative, Matiranga 4450, Bangladesh; russel_1204@hotmail.com

**Keywords:** Bengal slow loris, Pittachhara Forest, small- and medium-sized wild mammals, leopard cat, activity patterns, seasonal variation

## Abstract

The Chattogram Hill Tracts (CHTs) in Bangladesh have a rich biodiversity, hosting a wide range of wild mammal species, underscoring the importance of systematic monitoring focused on conservation efforts. This study aims to assess the diversity and abundance of small- and medium-sized wild mammal species in Pittachhara Forest in the CHTs. A comprehensive wildlife monitoring survey was conducted in this forest using strategically placed camera traps to identify the wild species diversity, and we evaluated the activity patterns and seasonal variations for the period of February 2023 to August 2024. The camera trap identified eight species of small- and medium-sized wild mammals: the Bengal slow loris, northern pig-tailed macaque, leopard cat, large Indian civet, common palm civet, crab-eating mongoose, northern tree shrew, and black rat. The activity patterns showed distinct temporal behaviors among these wild mammals, with nocturnal activity dominating for the Bengal slow loris and leopard cat, while the northern pig-tailed macaque exhibited diurnal activity. Seasonal variations demonstrated a significant difference in increased activity among most wild mammal species throughout the summer, particularly the large Indian civet and crab-eating mongoose, likely due to favorable environmental conditions, and decreased activity in the winter (*p* < 0.05). The findings suggest an intricate connection between species-specific behaviors and environmental factors that influence activity patterns, with increased activity in summer and a decrease in winter, indicating that summer conditions may enhance the movement and foraging behaviors of mammals. This study underscores the necessity for continuous biodiversity monitoring followed by conservation efforts in Pittachhara Forest to reduce the threats of habitat fragmentation, human disturbances, and inadequate protection, thus protecting the survival of endangered wild mammal species and maintaining the environmental harmony of this small, protected area.

## Simple Summary

The Chattogram Hill Tracts (CHTs) in Bangladesh support diverse wild mammal species, underscoring the need for conservation-focused monitoring. Camera traps in the Pittachhara Forest identified eight small- and medium-sized mammals, including the Bengal slow loris, northern pig-tailed macaque, and leopard cat. Activity patterns revealed the nocturnal behavior of species such as the Bengal slow loris, while the northern pig-tailed macaque was diurnal. Seasonal analysis showed significantly higher activity during summer, particularly for the large Indian civet and crab-eating mongoose, and reduced activity in winter (*p* < 0.05). This study highlights the need for ongoing monitoring and conservation to mitigate human impacts and ensure the survival of endangered species in this small, protected area.

## 1. Introduction

Bangladesh is strategically situated within the Indo-Burma biodiversity hotspot, serving as a vital transition zone for wildlife habitats in southeast Asia [1]. Bangladesh is home to significant natural regions, including the world’s largest mangrove forest known as the “Sundarbans”, as well as semi-evergreen to evergreen forests in the northeast (Habiganj and Moulvibazar districts of the Sylhet Division) and southeast, the Chattogram Hill Tracts (CHTs), and the greater Chattogram area. Previous studies have reported that the CHTs possess a diverse range of fauna, such as Asian elephants, gaur, dhole, tigers, Indo-Chinese clouded leopards, fishing cats, sambar deer, and hoolock gibbons [2,3,4]. However, the growing human population in Bangladesh exerts considerable pressure on ecosystems, including habitat fragmentation and resource exploitation, hence highlighting the urgent necessity for conservation to balance human needs with preserving biodiversity across the country, including in the CHTs [5,6].

The establishment of protected areas is essential in the global effort to reverse the decline of tropical forests and conserve wildlife biodiversity by limiting or reducing human activities in these regions [7,8,9]. To preserve biodiversity, the Bangladeshi government has spent decades establishing a network of protected areas, which features 20 national parks and 24 wildlife sanctuaries across the country, including in the CHTs [10]. However, despite national efforts and the designation of certain areas within the CHTs as protected, the region has experienced significant forest degradation in recent decades due to various factors, including shifts in agricultural practices, farming in forested areas, illegal logging, hunting, encroachment, and the conversion of natural forests into timber or industrial plantations [11,12]. This indicates that these challenges have hindered the execution of successful biodiversity conservation initiatives, leading to a substantial decline in wildlife species [13,14]. Therefore, it is essential to actively engage local communities, conservation organizations, researchers, and policymakers to protect biodiversity rather than passively relying on government policies. Mahfuz Ahmed Russel built a private protected area called the “Pittachhara Forest and Biodiversity Conservation Initiative (Pittachhara Forest)” to safeguard forest fragments in the adjacent area and preserve the existence of wildlife species in the central CHTs region. Before starting the initiative for the Pittachhara Forest, Mr. Russel was a buying manager in the United Kingdom. Recognizing the lack of fulfilment in this path, he abandoned most of his belongings and immersed himself in nature. Upon his return to Bangladesh, he left city life, and, with the assistance of childhood friends, he acquired forested land that was designated Pittachhara Forest. Initially dedicated to a sustainable, self-sufficient lifestyle, he was motivated to become a conservationist after witnessing the unsustainable shooting of wildlife by local communities of the CHTs, motivating him to take action [15]. Recently, a newspaper report highlighted Pittachhara Forest, underlining the challenges faced by its biodiversity to enhance public awareness and promote urgent measures for wildlife conservation and ecological balance in this protected area [15]. This report inspired us to conduct research in the region. However, no comprehensive scientific study has been carried out in Pittachhara Forest to date.

Camera trapping has emerged as a powerful tool for wildlife research, providing an unobtrusive method to monitor animal populations and behaviors [16]. Unlike conventional wildlife survey methods, camera traps function continuously under diverse environmental conditions, allowing for the collection of data on species’ presence, activity patterns, and interactions with their habitat [17]. Camera traps provide a significant advantage for documenting wildlife in comparison to alternative methods, such as direct observation, line transect surveys, or spoor tracking. However, detailed information on wild mammal species in Pittachhara Forest is limited. The forest serves as a protected area for many wild species including the northern pig-tailed macaque and many species of resident and migratory birds that have adapted to the region’s diverse forest environment [15]. This knowledge gap underscores the need for comprehensive research to understand the wild mammal species that inhabit this area and develop effective conservation strategies in the near future. Therefore, the aim of the current study is to gain a broader understanding of the present status of small- and medium-sized wild mammal species in Pittachhara Forest using a camera trap survey. To the best of our knowledge, this is the first camera trap survey in this forest.

## 2. Materials and Methods

### 2.1. Study Area

**Figure 1:**
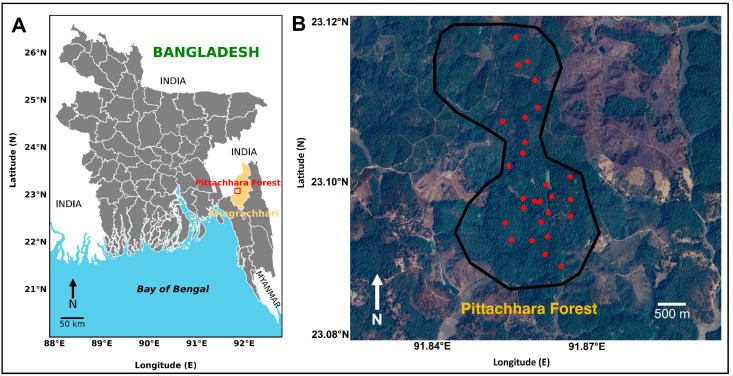
The geographic location of the studied area in Bangladesh: (**A**) a map of Bangladesh highlighting Pittachhara Forest (marked in the red box inside the yellow-highlighted area); (**B**) the area of Pittachhara Forest in which the camera trap survey was conducted. The red dots indicate the camera trap stations and the black line indicates the area of Pittachhara Forest.

Pittachhara forest is a 23-acre protected forest area located in East Khedachara Village, Belchhari Union, Matiranga Upazila, Khagrachari District, in the Chattogram Hill Tracts (CHTs), Bangladesh (Figure 1A). This region comprises the wider CHTs that are located very close to the Bangladesh/India border and are distinguished by their unique topography, such as undulating hills and dense woodlands. During the study period from February 2023 to August 2024, the mean annual precipitation exhibited a range of 2500 to 3000 mm; the average maximum and minimum temperatures were 36 °C and 13 °C, respectively; and the average humidity ranged from 75% to 95% (data obtained from https://www.accuweather.com/en/bd/, accessed on 30 August 2024). The high humidity levels and abundant rainfall support the growth of rich vegetation, thereby contributing to the forest’s remarkable biodiversity.

### 2.2. Camera Trap

We conducted the camera trap survey in Pittachhara Forest from February 2023 to May 2023 and from October 2023 to August 2024, deploying a total of ten Bushnell Trophy Cam HDs (119717CW and 119987M, China). The camera traps were deployed randomly on active wild animal paths such as areas of feeding and resting, valleys, streams, and bamboo groves. The average distance between camera traps was estimated to be 200–350 m. Each camera trap was attached to a tree between 1 and 1.5 m above ground level. The cameras were affixed to trees to provide a secure fit using a chain and placed inside an iron box to protect them from potential damage or tampering by wildlife or human interference or theft. A concerted effort was made to eliminate undergrowth foliage and twigs from the camera’s exposure range to maximize the acquisition of high-quality images of moving wild animals. The motion-sensor mode was utilized to take three consecutive photographs, which were then followed by a video recording that lasted for ten seconds. To maintain the integrity of our observations, we refrained from employing any bait or lure substances. Consequently, they were capable of capturing videos/images of wildlife with a minimal interframe delay of 10 s. The camera traps were in operation continuously for a duration of 24 h, and a comprehensive inspection was conducted over a period of 14–15 days to assess the memory card conditions, changing the memory cards, battery status, and overall functionality of the camera trap device. We relocated the camera traps monthly to ensure complete coverage of the study area and to capture a wide range of wildlife activities. A date and time stamp were affixed to every image that was shot in order to make the process of data organization more straightforward [18].

### 2.3. Data Analysis

We identified the wild animal species using previously published studies [19,20,21]. To focus and assess the efficacy of our camera trap wild mammal species inventory, we selected a subset of images that exclusively included small- and medium-sized wild mammals (weighing ≥ 0.5 kg). Furthermore, we consulted the expertise of field wildlife guides and experts from Pittachhara Forest to verify the identifications when necessary. All the videos and images were manually inspected to distinguish authentic wildlife species from fake triggers. To improve accuracy, the videos and images were evaluated separately among multiple team members, and, if needed, unreliable information was excluded from the file. We also excluded arboreal species and ground-dwelling avifauna due to the focus on small- and medium-sized wild mammals. The data from the camera traps were processed with Flexible Renamer ver. 8.4 and NeoFileInfolist ver. 1.4.1.0 software to compile raw observations. The raw data were then visualized using the Python package Matplotlib (version 3.9.2) to evaluate the species accumulation, activity patterns, and seasonal variations in Pittachhara Forest. This study categorized October to February as the dry winter season and April to August as the wet summer season for the analysis of seasonal variations in activity patterns. We assessed the significant difference between the seasonal variation in activity patterns utilizing the Mann–Whitney *U*-test (*p* < 0.05).

## 3. Results

**Figure 2:**
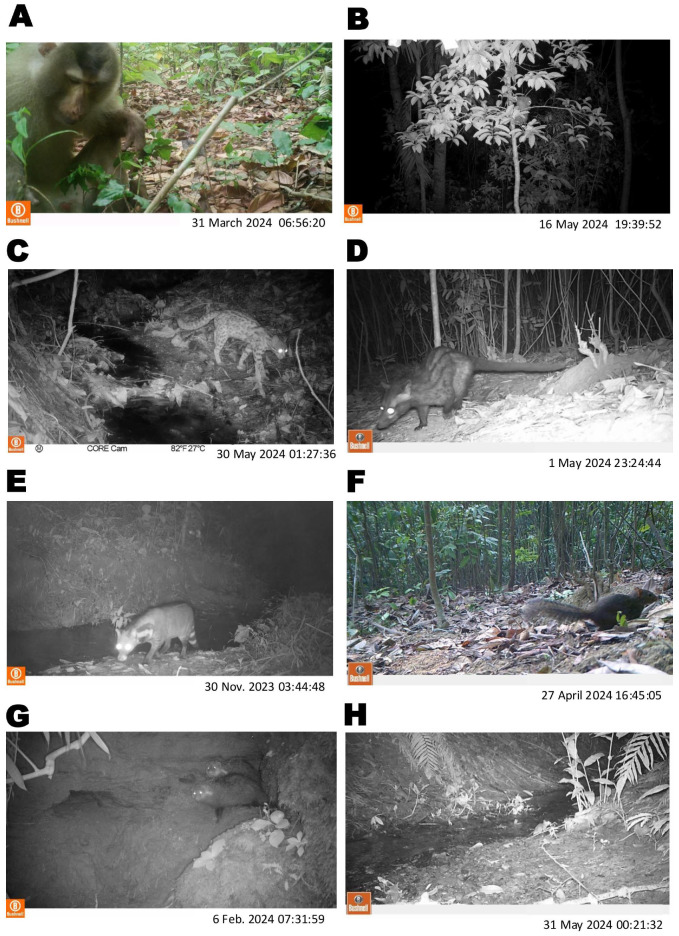
Camera trap images of small- and medium-sized wild mammal species that were identified in Pittachhara Forest, Bangladesh: (**A**) northern pig-tailed macaque; (**B**) Bengal slow loris; (**C**) leopard cat; (**D**) common palm civet; (**E**) large Indian civet; (**F**) northern tree shrews; (**G**) crab-eating mongoose; and (**H**) black rat.

The current study identified a total of 3465 camera trap data (videos and images) throughout the 27 camera trap areas in Pittachhara Forest from a total 216 camera trap nights. A total of 1313 captures documented various wild animal species in the forest, whereas the remaining 2152 videos and images did not consist of any wild animal species. This study recorded over 11 different species of wild animals, including mammals and avian species. Among the identified wild animal species, three were small-sized wild mammals, namely, the crab-eating mongoose (*Herpestes urva*) (*n* = 50), northern tree shrew (*Tupaia belangeri*), and black rat (*Rattus rattus*) (*n* = 21), and five were medium-sized wild mammals, namely, the Bengal slow loris (*Nycticebus bengalensis*) (*n* = 1), northern pig-tailed macaque (*Macaca leonina*) (*n* = 317), leopard cat (*Prionailurus bengalensis*) (*n* = 4), large Indian civet (*Viverra zibetha*) (*n* = 3), and common palm civet (*Paradoxurus hermaphroditus*) (*n* = 8) (Figure 2 and Table 1). The camera trap data also showed a variety of non-target wild species and other activities such as greater horseshoe bats (*n* = 6), red jungle fowl (*n* = 12), a pheasant (*n* = 1), unidentified bird species (*n* = 448), human activities (*n* = 38), and dogs (*n* = 10) in Pittachhara Forest. Table 1 also indicates that the northern pig-tailed macaque was the most abundant species followed by the crab-eating mongoose and the northern tree shrew in the forest. The less frequently observed species included the Bengal slow loris, leopard cat, and large Indian civet. Additionally, avian species were recorded including the pheasant and red jungle fowl.

**Figure 3:**
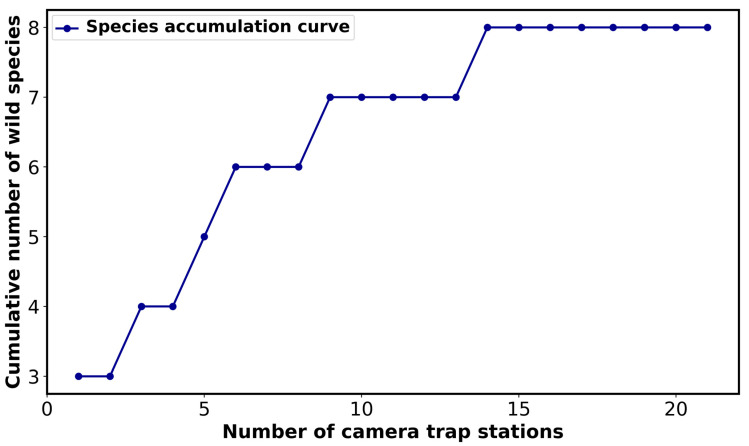
Species accumulation curve for wildlife in Pittachhara Forest. The species accumulation curve illustrates the association between the total number of camera trap stations (horizontal axis) and the total number of species identified (vertical axis). The blue line shows the accumulation of species when more camera trap locations were investigated. Each point on the line represents the total number of species identified at that particular number of camera trap locations.

The species accumulation curve demonstrates that wild species were identified at 22 out of the 27 camera trap stations in Pittachhara Forest (Figure 3). The other five camera stations did not capture any wildlife and instead produced blank images or videos. The results show the effectiveness of the sampling effort in capturing the majority of the species present in Pittachhara Forest. The curve shows a gradual plateau, indicating that most wild species were consistently distributed across the sampled locations. Due to our emphasis on small- and medium-sized wild mammals, we omitted the other species and objects from further investigation.

**Figure 4:**
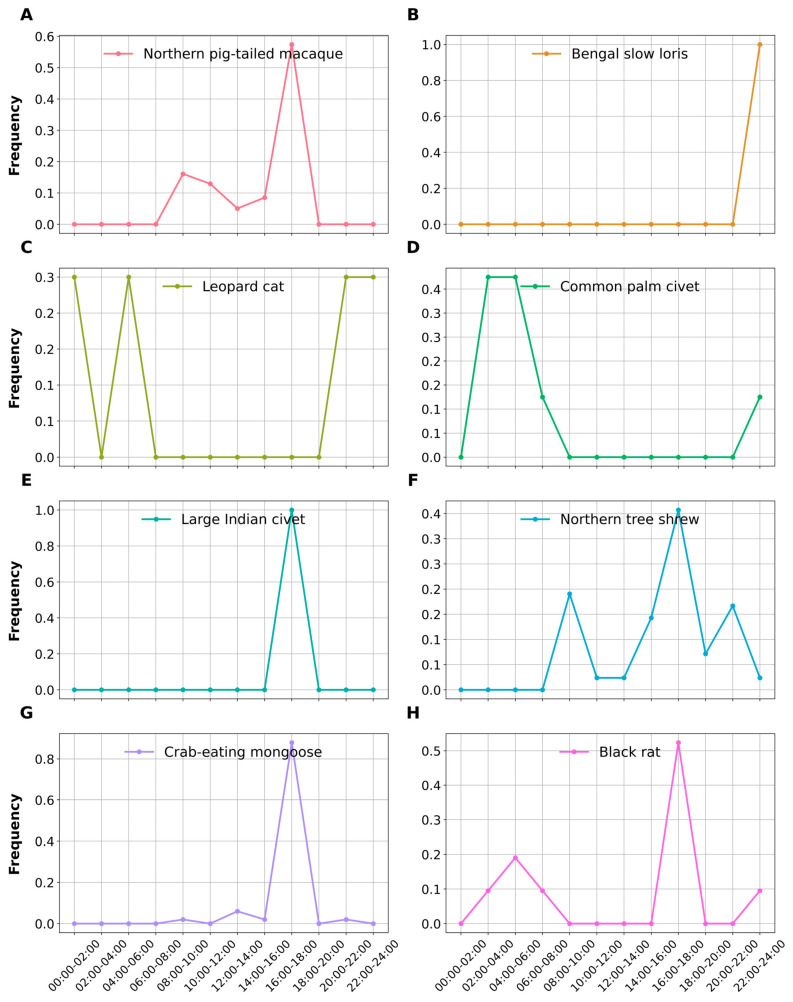
The activity patterns of eight small- and medium-sized wild mammal species were illustrated based on camera trap data from Pittachhara Forest. The y-axis represents the frequency of detected wild mammal species (log transformed), while the x-axis indicates the 24 h time period divided into 2 h intervals. (**A**) northern pig-tailed macaque; (**B**) Bengal slow loris; (**C**) leopard cat; (**D**) common palm civet; (**E**) large Indian civet; (**F**) northern tree shrews; (**G**) crab-eating mongoose; and (**H**) black rat.

The activity patterns of different wild species observed in Pittachhara Forest were analyzed (Figure 4), illustrating the temporal activity over a 24 h period for different wild species. Of the wild small- and medium-sized wild mammal species, the Bengal slow loris exhibited its highest activity levels in the late evening (22:00–24:00), whereas the common palm civet was primarily active during the early morning (02:00–04:00) and experienced another notable peak around midday (12:00–14:00). The crab-eating mongoose and northern pig-tailed macaque exhibited increased activity levels between 16:00 and 18:00, with the macaque also displaying a smaller secondary peak in activity at 06:00–08:00. The large Indian civet demonstrated significant activity in the early morning (04:00–06:00) and again around 18:00. The leopard cat exhibited increased activity during the late afternoon and early evening, specifically between 16:00 and 20:00, due to their nocturnal nature. The northern tree shrews and black rat exhibited moderate activity during the early and late afternoon.

**Figure 5:**
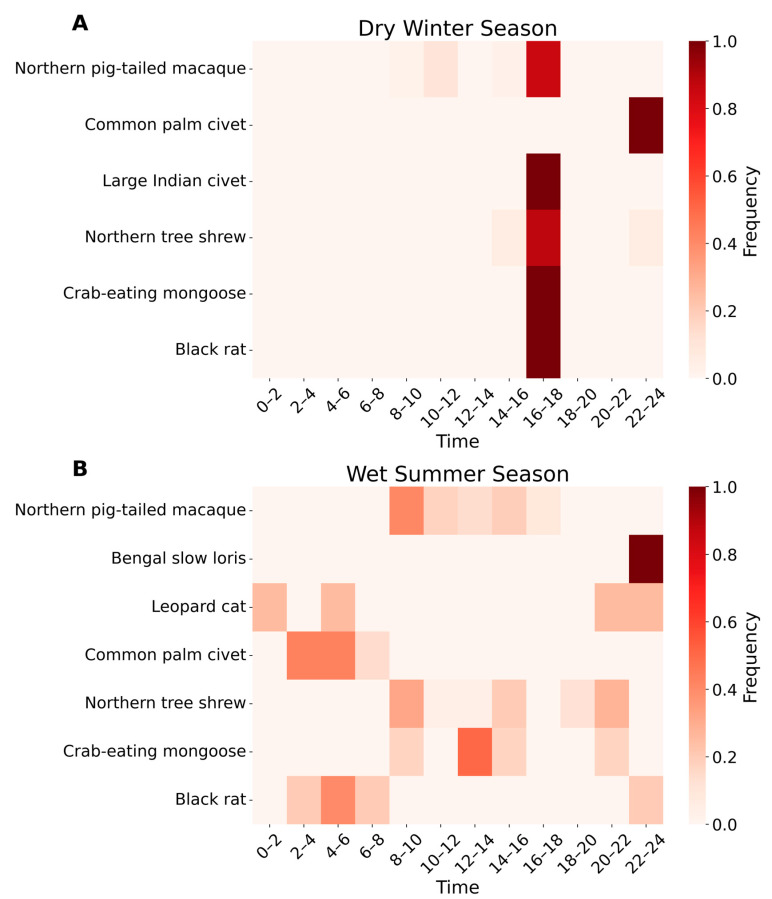
Comparative seasonal activity patterns of small- and medium-sized wild mammals in Pittachhara Forest during (**A**) the dry winter season and (**B**) the wet summer season. The rows represent different species, while the x-axis indicates time intervals. The activity frequency is log-transformed, and the intensity of the color corresponds to the relative frequency of activity (darker shades represent higher activity levels, while lighter shades represent lower activity levels). The red gradient scale on the right shows the log-transformed frequency values ranging from 0.0 (minimum) to 1.0 (maximum).

The seasonal activity patterns of small- and medium-sized wild mammal species are represented in the heat maps (Figure 5). The results showed significant differences in the activity patterns of small- and medium-sized wild mammal species between the dry winter and the wet summer seasons (*p* < 0.05). The results also identified distinct variations in response to the changing environmental conditions of Pittachhara Forest in the hill tract region. During the dry winter season, small- and medium-sized mammal activity was concentrated around 16:00–18:00, with species such as the northern pig-tailed macaque, common palm civet, and crab-eating mongoose showing peak activity, while others, such as the northern tree shrew and black rat, exhibited minimal activity. In contrast, during the wet summer season, small- and medium-sized mammal activity significantly increased and was distributed throughout the day and night (*p* < 0.05). Crepuscular patterns were observed in species such as the crab-eating mongoose and leopard cat, with activity peaks in the early morning and late afternoon. The large Indian civet showed heightened activity, particularly in the early evening, while the northern tree shrew exhibited increased activity throughout the day, peaking in the morning and afternoon.

## 4. Discussion

The CHTs in Bangladesh host a rich diversity of wildlife, including several small- to large-sized wild mammals [2,21,22,23,24,25]. The mammalian fauna in the CHTs is currently confronted with significant problems, primarily attributable to elevated deforestation rates and illegal hunting [4,26,27,28]. The present study confirmed the presence of eight small- and medium-sized wild mammal species in Pittachhara Forest in the CHTs. Studies from other regions have found relatively few wild mammal species within large, protected areas. For example, 17 wild mammal species were identified within the 1985 km^2^ of the Namdapha Tiger Reserve in India, 35 species were documented across 17,373 km^2^ of the Huay Kha Khaeng Wildlife Sanctuary in Myanmar, and 26 species were recorded within the 1122 km^2^ of Tabin Wildlife Reserve in Malaysia [29,30,31]. In our study, the identification of a small number of wild mammal species in Pittachhara Forest is likely due to its status as a small, protected area, compounded by surrounding forest degradation, human disturbances, and potential limitations of our camera survey coverage and methodology.

We documented small- and medium-sized wild mammal species of significant conservation concern, including the endangered Bengal slow loris and two globally vulnerable species, namely, the leopard cat and the northern pig-tailed macaque. These wild mammal species are indeed present in Pittachhara Forest, contributing to its biodiversity and highlighting the ecological importance of this region for the conservation of these threatened species. The identification of the Bengal slow loris for the first time in Pittachhara Forest during the camera trap survey further underscores the significance of this region. As the Bengal slow loris primarily lives in the treetops, being nocturnal and cryptic in nature, detecting this species is unusual, making its identification in Pittachhara Forest particularly noteworthy. This area retains some significant ecological features that are essential for its survival, such as dense forest cover and suitable tree canopy. Previous sightings of the Bengal slow loris have been sporadic in other parts of Bangladesh, such as the northeastern Sylhet region [25,32]. However, such sightings are rare, highlighting the challenges in detecting this elusive species in various regions of the country. The current study also confirmed the presence of another primate species, the globally vulnerable northern pig-tailed macaque, in Pittachhara Forest. This primate has a semi-terrestrial lifestyle, inhabiting both forest interiors and edges. The abundant presence of northern pig-tailed macaques in the forest indicates that this area likely offers suitable habitats with adequate resources, such as food and shelter, to support the species’ survival and well-being. Previous studies have documented the northern pig-tailed macaque species in protected and unprotected forests in other areas such as Lawachara National Park, Satchari National Forest, and Teknaf Wildlife Sanctuary [20,21,25,33,34,35]. However, their existence is increasingly threatened and believed to be declining inside and outside of protected areas due to habitat degradation, food scarcity, illegal hunting, and human encroachment.

The current study confirmed only one cat species in Pittachhara Forest, the leopard cat, which has experienced a gradual population decline in the CHTs. Previous studies documented several wild cat species in the CHTs and Sylhet regions, including the tiger (*Panthera tigris*), leopard (*Panthera pardus*), fishing cat (*Prionailurus viverrinus*), clouded leopard (*Neofelis nebulosa*), marbled cat (*Pardofelis marmorata*), Asiatic golden cat (*Catopuma temminckii*), and leopard cat (*Prionailurus bengalensis*) [14,21,23,34,36]. The disparity can arise from variations in habitat preferences and also from the precise positioning of our camera traps. Nevertheless, the camera traps employed in our study failed to record these species (except the leopard cat), presumably indicative of the declining numbers of these species, habitat differences, and the hunting challenges posed by the ethnic locals. Interestingly, our study reported two civet species in Pittachhara Forest, the large Indian civet and the common palm civet. Previous studies reported that the large Indian civet, the heavier of the two species, is predominantly terrestrial and nocturnal, and the common palm civet is arboreal, but it has also been seen foraging on the ground [21,34,36]. Our camera traps did not capture various species in Pittachhara Forest that have been conclusively recorded at different areas in the CHTs and other regions of Bangladesh by prior research. The species include the Asiatic wild dog or dhole (*Cuon alpinus*), Chinese pangolin (*Manis pentadactyla*), Asiatic black bear (*Ursus thibetanus*), Malayan giant squirrel (*Ratufa bicolor*), Phayre’s leaf monkey (*Trachypithecus phayrei*), hog badger (*Arctonyx collaris*), wild boar (*Sus scrofa*), Indian porcupine (*Hystrix indica*), barking deer (*Muntiacus muntjak*), and Asian elephant (*Elephas maximus*) [3,14,20,21,23,36,37]. The absence of these wild species may be indicative of Pittachhara Forest’s small ecological area, the habitat not being suitable for large wild mammals, localized extinctions, habitat fragmentation, and significant changes in the wild mammal community likely driven by factors such as deforestation, poaching, and agricultural expansion, which highlight an urgent necessity for targeted conservation efforts to protect the identified species and their habitats.

The daily activity patterns of small- and medium-sized wild mammal species vary and may be influenced by their internal biological clock along with other environmental and ecological factors [38]. In our study area, we observed that the Bengal slow loris, leopard cat, and civet species were exclusively nocturnal, which aligns with the findings of previous reports [21,34,39,40]. In contrast, other wild mammals, such as the crab-eating mongoose, were predominantly crepuscular. However, their presence was influenced by human activity, including livestock, as humans, livestock, and the crab-eating mongoose all shared the same walking paths [41]. The current study shows that the northern pig-tailed macaque is a diurnal primate, signifying that its activity is restricted to daylight hours. The diurnal behavior of the northern pig-tailed macaque facilitates foraging, socialization, and other vital activities during optimal visibility, hence minimizing resource conflicts with nocturnal species [37,42,43]. Previous studies also indicated that diurnal primates, such as macaques, are frequently more susceptible to predators and human interference, exacerbating the conservation challenges they face, especially in fragmented habitats in which their daytime activities can increase their vulnerability to threats [20,23,25,34,44,45]. Furthermore, daily activities may mitigate competition among associated wild mammals, facilitating more successful coexistence of species sharing in the same habitat by diminishing direct resource rivalry. The results revealed significant differences in the seasonal activity peaks of small- and medium-sized wild mammals in Pittachhara Forest. Despite the absence of year-round data from our camera traps, we noted significant seasonal fluctuations in the activity patterns of small- and medium-sized wild mammals, particularly contrasting the summer and winter seasons. These seasonal variations in activity patterns may be closely related to the availability and quality of food resources across different seasons in Bangladesh. The activity level of small- and medium-sized wild mammals exhibited a marked increase during the wet summer season, while dry winter season activity levels were significantly lower and peaked only at specific times compared to the more sustained activity observed in summer [20,34,46]. This reduction in activity and movement during the colder months is likely an energy conservation strategy, allowing the animals to minimize energy expenditure. Additionally, they may spend more time digesting lower-quality food, which is often more prevalent in winter, further contributing to the decrease in overall activity [39,47]. This study emphasizes the urgent need for conservation measures in Pittachhara Forest, such as increasing monitoring to combat poaching, restoring degraded habitats, and designating the forest as a protected area to preserve its unique biodiversity. While the recorded wild mammal species in this study likely do not represent the region’s full biodiversity, due to the short survey period and species-specific behaviors, the use of camera traps has proven effective in estimating species abundance. Future research could benefit from extended camera trap deployment, focusing on small- and medium-sized mammals, arboreal species, and semi-aquatic organisms. Long-term conservation will require landscape-scale ecosystem management, regular monitoring, stronger protection efforts, and local community involvement.

## 5. Conclusions

In conclusion, this is a preliminary piece of research that highlights the monitoring of small- and medium-sized wild mammal species in Pittachhara Forest. This study provides essential insights into the diversity, densities, and activity patterns of small- and medium-sized wild mammal species in this area. It offers a scalable framework that enhances camera trap analysis from single-species to multi-species evaluations, contributing to the conservation efforts in Pittachhara Forest. Notably, this study identified eight small- and medium-sized wild mammal species in the forest. The activity patterns of these species were quite diverse, in accordance with seasonal variation. This study underscores the necessity for intensified efforts in the monitoring of these species within Pittachhara Forest. The collected data are crucial for comprehending the ecological functions of these obscure species; however, enhanced survey methodologies and cooperation among researchers and conservation organizations will be vital to achieve a more precise evaluation of their behavioral patterns and conservation requirements in this area. This camera trap survey was conducted in Pittachhara Forest for the first time. Future research should prioritize deploying additional camera traps across diverse routes, habitats, and areas, supported by advanced species occupancy models for deeper insights.

## Figures and Tables

**Table 1:** The camera traps identified terrestrial wild animals and other non-target objects in Pittachhara Forest, Bangladesh.

Name	Order	Family	Scientific Name	Total Count	Relative Abundance	Species Status	
						National	IUCN
Northern pig-tailed macaque	Primates	Cercopithecidae	*Macaca leonina*	317	0.330	EN	VU
Bengal slow loris		Lorisidae	*Nycticebus bengalensis*	1	0.001	EN	VU
Common palm civet	Carnivora	Viverridae	*Paradoxurus hermaphroditus*	8	0.010	LC	LC
Large Indian civet			*Viverra zibetha*	3	0.004	NT	LC
Crab-eating mongoose		Herpestidae	*Herpestes urva*	50	0.050	NT	LC
Leopard cat		Felidae	*Prionailurus bengalensis*	4	0.005	NT	LC
Northern tree shrew	Scandentia	Tupaiidae	*Tupaia belangeri*	42	0.040	LC	LC
Black rat	Rodentia	Muridae	*Rattus rattus*	21	0.020	LC	LC
Greater horseshoe bat	Chiroptera	Rhinolophidae	*Rhinolophus ferrumequinum*	6	0.010	LC	LC
Pheasant	Galliformes	Phasianidae	*Phasianus colchicus*	1	0.350	EN	LC
Red jungle fowl			*Gallus gallus*	12	0.010	EN	LC
Human activities			-	38	0.040	-	-
Dog			-	10	0.010	-	-
Unidentified bird species			*-*	448	0.470	-	-

EN, endangered; NT, near threatened; LC, least concern; VU, vulnerable.

## Data Availability

The data presented in this study are available within the article.
